# “The Earth Is Becoming a Coronavirus”: Children’s Perceptions, Knowledge, and Experiences of the COVID-19 Pandemic as Presented in Their Drawings

**DOI:** 10.1177/10497323251334247

**Published:** 2025-04-26

**Authors:** Sofia Gjertsson, Maria Thell, Anna Sarkadi

**Affiliations:** 1 Child Health and Parenting (CHAP), Department of Public Health & Caring Sciences, 174463 Uppsala University, Uppsala, Sweden

**Keywords:** children’s drawings, children’s experiences, health literacy, pandemic, COVID-19, visual analysis, content analysis

## Abstract

Younger children’s voices are often overlooked in research and policy, yet their perceptions and experiences are crucial in understanding their needs in planning responses to crisis, such as the COVID-19 pandemic. Existing studies on children’s experiences of crises often rely on adult perspectives or are adult-led; a more child-centric approach could be to analyze children’s drawings. The aim of this study was to explore Swedish 7- to 11-year-old children’s experiences and knowledge of the COVID-19 pandemic, as expressed in their drawings. Drawings from *N* = 454 children with accompanying texts, from various Swedish municipalities with different socio-economic profiles, were analyzed using a combination of semiotic visual analysis and content analysis. Three main themes emerged: (a) *Fun, friends, and freedom are cancelled* which pointed to societal changes as a result of the pandemic that impacted the children’s own lives, causing high levels of frustration; children saw contrasts between what had been, what is, and what is to come; (b) *The world is all upside down*, in which the children highlighted their understanding of the virus, how it has changed and impacted the world, as well as existential reflections of their lives and surrounding environment; and (c) *The Virus: evil, scary, and dangerous, but masks and sanitizer can help* showed the children’s understanding of the pandemic as a global event and showed high level of health literacy related to COVID-19. Children were very perceptive and astute to the societal issue at hand. Despite Sweden’s relatively lenient measures, the pandemic significantly affected their lives and autonomy.

## Introduction and Background

The COVID-19 pandemic was a crisis where knowledge, or rather lack of knowledge, about the virus took center stage. Never before has the state of knowledge about the clinical manifestation, spread of an infectious disease, and the effect of protective measures advanced so quickly and in front of the public eye. Children and young people became, partly involuntarily, consumers of information and media discussions about the virus and the disease. Our research team previously found that worry was common among children (age 4–12 years old) early in the pandemic (Sarkadi et al., 2021). Children also described negative social effects, such as not being able to see grandparents and missing out on recreational activities, which is consistent with UNICEF’s (UNICEF Sverige, 2020) and the Children’s Ombudsman’s surveys (Barnombudsmannen, 2021). Previous studies also found that children (age 5–13 years old) with severe disabilities were particularly vulnerable due to social isolation and loneliness (Fäldt et al., 2022). In Spain, children (*N* = 250, age 3–12 years) also described the negative feelings of loneliness, boredom, and fear, especially at younger ages. However, children have also described some positive aspects of the pandemic such as spending more time with their parents (Idoiaga et al., 2020).

### The Use of Children’s Drawings in Research

Most studies on children’s experiences and knowledge on societal disasters and crises, such as the pandemic, have used parents or other adults as sources. The few studies that directly explored children’s perspectives have had a linguistic bias, limiting children’s stories to what they can express in words (Aronsson, 2022; Walker et al., 2009). Furthermore, it is only in the last decade that children have been more involved in disaster risk reduction efforts and in research (Amri et al., 2018). A previous study by Fothergill and Peek (2015) noted that children often express their emotions and knowledge about disasters through various art forms such as drawing and other forms of performance (Fothergill & Peek, 2015).

Hence, one way of approaching the experiences and knowledge of younger children is to examine what they express in drawings. Studying children’s drawings has a long tradition within research and is considered a child-centered approach and enables access into the child’s world (Aronsson, 2022). Traditionally, children’s drawings were mainly used as a non-projective means of measuring developmental or intellectual maturity, or used in clinical tests for psychological diagnoses of personality or emotional difficulties (Pridmore & Bendelow, 1995). Drawings have since been used in health education and more recently in understanding children’s perceptions of health and illness (Piko & Bak, 2006; Pridmore & Bendelow, 1995). Through the use of drawings, Piko and Bak were able to gain an understanding in how young children (8–11 years old) perceived health and health promotion, where cleaning rituals were a means for children to avoid getting common infectious diseases such as colds. Children also expressed great self-awareness regarding their own health (Piko & Bak, 2006).

Although an effective tool, it should be noted that, to gain the best understanding of a child’s drawing, it is often preferred that it be accompanied with written or oral accounts by the child (Pridmore & Bendelow, 1995; Ribeiro & Silva, 2020). Pridmore and Bendelow further argue that when utilizing children’s drawings as a research method, the aim should be to treat the children as subjects rather than objects, as they argue that traditionally, research has been *on* rather than *with* children, using adult categories and data (Pridmore & Bendelow, 1995).

#### Children’s Drawings of the COVID-19 Pandemic

There have been a few studies on children’s knowledge and experiences, utilizing qualitative interviews, survey responses, and children’s drawings of the COVID-19 pandemic where the results have generally covered three main areas. They are (a) representations and depiction of the virus; (b) reactions to the effects of the COVID-19 pandemic, including both direct and indirect consequences; and finally, (c) health literacy and access to information.

#### Representations and Depiction of the Virus

One of the main features presented in drawings of the COVID-19 virus across different countries was the emphasis that children have a good understanding of the appearance of the COVID-19 virus, often drawing realistic pictures of what the virus particle looks like. Children’s illustrations of the COVID-19 virus also often included anthropomorphic and fantasy elements, even at older ages, but the anthropomorphic images had clear links to realism and the “actual” appearance of the virus (Bonoti et al., 2021; Martinerie et al., 2021; Rydström et al., 2022; Sarkadi et al., 2022; Tishelman et al., 2022). In Spain, authors found that children (age 6–12 years old) often illustrated COVID-19 based on the images circulated in the media, often including elements of anthropomorphism (Idoiaga Mondragon, Eiguren Munitis, Berasategi Sancho, & Ozamiz Etxebarria, 2022). Similarly, in Sweden, children’s (age 4–6 years old) drawings demonstrated young children’s understanding of the “invisible enemy,” combining realistic parts of the virus particle with fantasies of the “virus monster” that had to be fought, sometimes tangibly by the children themselves (Sarkadi et al., 2022).

#### Reactions to the Effects of the COVID-19 Pandemic

The literature reports that children were scared of the COVID-19 virus and of their loved ones becoming infected, the most common emotion. Studies also found that children were worried and felt guilty about potentially infecting someone (Bonoti et al., 2021; Idoiaga et al., 2020; Idoiaga Mondragon, Eiguren Munitis, Berasategi Sancho, & Ozamiz Etxebarria, 2022; Idoiaga Mondragon, Eiguren Munitis, Berasategi Sancho, Picaza Gorrotxategi, et al., 2022). Studies from Spain (age 6–12 and 3–14 years old), Italy (age 5–12 years old), and France (age 5–17 years old) also found that children due to expressed reluctance to spend more time at home and frustration not being able to go outside, or to school, In addition, they described sadness about not being able to socialize, hug friends, and see grandparents (Cornaggia et al., 2022; González-Calvo et al., 2022; Idoiaga Mondragon, Eiguren Munitis, Berasategi Sancho, & Ozamiz Etxebarria, 2022; Idoiaga Mondragon, Eiguren Munitis, Berasategi Sancho, Picaza Gorrotxategi, et al., 2022; Martinerie et al., 2021).

#### Health Literacy and Access to Information

Health literacy consists of the ability and skills individuals have and need to understand, access, evaluate, and use health information and services to make decisions to promote good health. Addressing child health literacy is especially important to ensure long-term good health outcomes (Velardo & Drummond, 2017). Velardo and Drummond further argue that promoting health literacy at different developmental stages of children and adolescents is crucial as children gain more and more independence as they grow into adolescents and adulthood (Velardo & Drummond, 2017).

Health literacy linked to the COVID-19 pandemic and children’s ability to understand information from schools, media, and adults was common across the literature. Shown by Bray and colleagues in a study with data from the United Kingdom, Brazil, Sweden, Australia, Spain, and Canada, children aged 7–12 years had high health literacy on the topic (Bray et al., 2021). Children overall showed high levels of health literacy related to COVID-19, including transmission routes and the importance of hand hygiene and maintaining social distancing (Rydström et al., 2022; Sarkadi et al., 2022; Thompson et al., 2021; Tishelman et al., 2022). Knowledge of restrictions and new rules, how the disease spreads, and its characteristics was common. Children in Sweden (age 7–12 years old) also reported that they knew where to find information about the virus (Rydström et al., 2022).

#### Rationale

Exploring children’s perceptions and knowledge of the COVID-19 pandemic in Sweden is particularly interesting, as the Swedish response to the COVID-19 pandemic had significantly less mobility restrictions, school closures, and lockdowns in comparison to countries where most of the existing literature is situated. Little is still known about the experience of and knowledge about the COVID-19 pandemic among children aged 7–11 years, who resided in Sweden during the pandemic.

Using child-centered methods for data collection is still not the norm within child health research. More often, linguistic or written forms of data are collected, limiting the kind of information that children can express. While previous studies in Sweden have utilized similar methods (Sarkadi et al., 2022; Tishelman et al., 2022) (*N* = 94 and *N* = 187, age 4–6 years old and 13–15 years old), the sample in this study is significantly larger. Understanding children’s perceptions, experiences, and knowledge of the COVID-19 pandemic is central in furthering the work of child-centered disaster risk reduction, in order to understand how children are affected and what they retain from a societal crisis.

We have previously published an article on 4- to 6-year-old children’s experiences of the COVID-19 pandemic with data from Swedish Children’s Picture Archive (SBBA) (Sarkadi et al., 2022). We wanted to see how older children, attending school, experienced and understood the COVID-19 pandemic and were contracted by the Swedish Public Health Agency to do so, as part of a wider national report on the indirect effects of the pandemic.

## Aim and Purpose

The aim of this study is to explore Swedish 7- to 11-year-old children’s experiences and knowledge of the COVID-19 pandemic, as expressed in their drawings.

This study also aims to explore the feasibility, usefulness, and appropriateness of analyzing children’s drawings as data in child-centered health research.

## Methods

### Material

The material in this study consists of drawings from 7- to 11-year-old children, collected by the SBBA during the COVID-19 pandemic, between April 2020 and February 2021 (Barnbildarkiv, 2020).

The archive’s pandemic collection consists of over 1200 images in total, spanning from children aged 4–18 years old, and is open for researchers to use. Any identifying information, such as the child’s name, was removed prior to analysis.

SBBA’s collection of drawings also included written texts by the children that they could either write themselves or dictate to an adult. They were allowed to write whatever they wanted, but SBBA included four questions that the children could use as inspiration for both the drawings and for the texts. The questions are listed below:

(1) How does it feel?
(2) How does it look?
(3) What is different now?
(4) Is there anything else you would like to describe?

**Figure 1.**
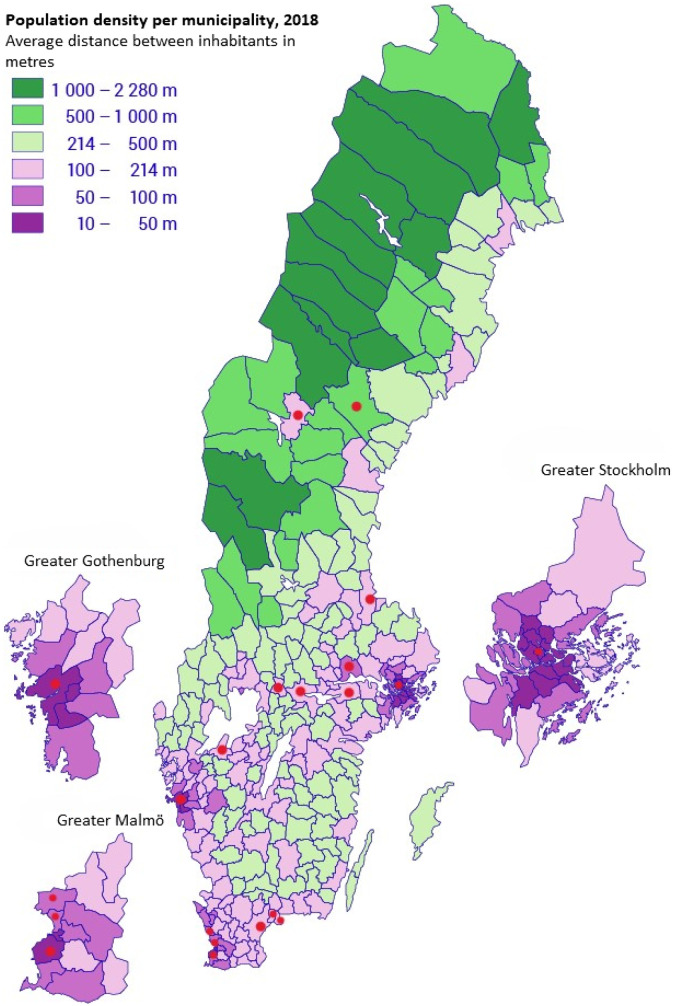
Map of included municipalities by population density. Copyright: Statistics Sweden.

SBBA sent out a call for drawings across the country, to schools, extra-curricular activities and clubs, on social media, and local news outlets. This was in order to get as broad of a reach as possible; however, most of the collected drawings were drawn and collected in schools in a classroom-setting. The 16 included municipalities are marked with a red dot on the map of Sweden (Figure 1).

Although no individual socio-economic data was collected, there is a great deal of information about the included municipalities which have a good mix between larger and smaller cities as well as urban and more rural areas. The represented municipalities are spread across Sweden and differ in median income, unemployment rates, levels of education, and the percentage of foreign-born residents (Table 1). This gives an indication of the socio-economic context at a municipal level in which the data was collected.

**Table 1.** Included Municipalities With Characteristics.

Parameters/Location	Median Income in SEK	Inhabitants Living on Benefits in %	Highly Educated Inhabitants in %	Unemployment in %	Foreign Born in %
Sweden	329,268	11.7	33.7	6.4	20.5
Bromölla	304,666	14.8	19.3	8.5	16.7
Eskilstuna	300,485	16.9	26.2	10.9	26.8
Gävle	322,324	15.1	30.2	8.9	16.5
Göteborg	336,000	11.5	42.2	6.9	29.6
Karlskoga	315,999	14.1	22	6.1	16.5
Kristianstad	309,227	15.8	29.4	9.3	19.9
Kävlinge	367,680	9.3	35.3	4.4	11.7
Linköping	325,624	10.9	43	5.7	18.7
Lomma	425,526	6.4	55.4	3.3	10.4
Malmö	300,782	14.1	39.2	12.1	35.8
Sollefteå	284,604	14.5	21.3	6.3	12.1
Solna	394,050	6.4	52.2	4.5	34
Sölvesborg	309,219	13.1	23.6	5.9	12.1
Västerås	329,955	13.7	34.1	8	24.7
Örebro	317,038	12.5	35.5	7.3	19.8
Östersund	318,100	12	35.5	4.6	10.7

(Ekonomifakta; StatisticsSweden; Statistikdatabasen).

### Inclusion Criteria

The study used two inclusion criteria: the first was that the picture had to be drawn by a child aged 7–11 years old and the second that the picture had to have accompanying text provided by the child. The reason for the latter was that we wanted to use the combination of images and text in the analysis, as reported in a previous study (Sarkadi et al., 2022). Therefore, all pictures that lacked the age of the child and/or accompanying text were excluded.

In the age group 7–11 years old, there were a total of 568 images in the SBBA archive. Of the 568 images, all were reviewed to ensure that they met both inclusion criteria as described above. After exclusion, 454 images (80%) remained and were included in the study.

### Analysis

The drawings were analyzed using NVivo, a qualitative analysis software where both the drawings and its accompanying texts were analyzed. The analysis was a combination of semiotic visual analysis (van Leeuwen & Jewitt, 2001) and qualitative content analysis (Graneheim & Lundman, 2004) and followed four steps.

The *first step* of the analysis was to examine the *denotation* of the images, that is, what the images represent and how. In this step, the shapes, colors, figures, centered objects, facial expressions, etc., were described (van Leeuwen & Jewitt, 2001, pp. 94–96). Here, pressure with the pen, size of objects, and their colors were taken into specific consideration. Colors, pen-pressure, and object size can give specific insight into the importance of difference object within the drawing composition. Hence, significant time was spent by reviewing these aspects in the initial step of the analysis. The presence of smaller objects was also recorded. In a few instances, the accompanying text was used at this stage, only to provide “pointers,” as surrounding text specifies depicted motifs (Aronsson, 1997, pp. 33–37; van Leeuwen & Jewitt, 2001, p. 96). In this study, this was rarely needed, but an example of how it was used can be seen in Figure 11: from the text “It’s a battle between the coronavirus and hand sanitizer,” researchers could understand that the COVID-19 virus was the black and red creature on the right side of the drawing, and the hand sanitizer was the blue creature on the left. This generated codes such as “blue hand sanitizer with sword” and “black and red virus particle with crossbow,” giving extra insight into the drawing; however, this was rarely used.

In the *second step*, the images were sorted into categories representing their manifest content. Categorization of the manifest content built on a level of generality (van Leeuwen & Jewitt, 2001, p. 95). Further, perspective of the image was also taken into consideration, such as central perspective, bird perspective, portrait perspective, and mixed perspective to name a few. Some images were sorted into more than one visual category if they had multiple motifs, for example, in Figure 11, there were two categories: “virus as a creature” and “fighting COVID-19.” Significant time was spent on steps 1 and 2 of the analysis.

In the *third step*, manifest content analysis (Graneheim & Lundman, 2004) of the texts accompanying the images was conducted. The texts were also analyzed using NVivo. This involved finding meaning units and condensing them into initial codes, and then collecting similar codes into categories. For example, the meaning unit “It’s a battle between the coronavirus and hand sanitizer” became the code “fighting COVID-19,” that later fell into the sub-theme *Children’s depictions of COVID-19*.

The *fourth step* was an interpretation, *connotation*, which involved a latent analysis of the representation and meaning of both the image and text content. This was done using a combination of semiotic visual and content analysis where the initial codes from both drawings and written texts were reviewed again and sorted into categories, sub-themes, and finally themes, based on the broader concepts, ideas, and values represented, which formed the latent content of the data (Graneheim & Lundman, 2004; van Leeuwen & Jewitt, 2001, pp. 96–97). To take the example above, Figure 11 showing a fierce fight between the virus as a creature and the hand sanitizer as a war hero was denotated as an image of a battle; the attached text generating the code “fighting COVID-19” fell under the sub-theme *Children’s depictions of COVID-19*, which then contributed to generate the connotated theme: *The Virus: evil, scary, and dangerous, but masks and sanitizer can help*, containing three sub-themes (see Table 2). Further, the texts could also reinforce of strengthen what was happening in the drawing.

**Table 2.** Themes and Sub-Themes.

Themes	Sub-Themes
Fun, friends, and freedom are cancelled	(Strong) reactions to prohibitions
	Contrasts
The world is all upside down	The whole world is affected
	Existential thoughts
The Virus: evil, scary, and dangerous, but masks and sanitizer can help	Children’s depictions of COVID-19
	COVID-19 leads to illness and death
	Means of protection and reduction of transmissions

Three researchers conducted the analysis in parallel with discussions after each step to ensure that the analysis was carried out consistently throughout the study, while leaving a transparent decision trail.

### Pilot Study

In order to test the method and to see what trends were present in the children’s drawings, a pilot study was carried out on 50 entries, representing approximately 10% of the included drawings. The selection of images was randomized, although maintained an even distribution across the included age groups and gender. The analysis process followed the same procedure as described above, with the exception of the final step, the latent analysis, which was deemed unsuitable for the pilot phase, as researchers wanted to maintain an open mind about potential new categories to generate themes that could emerge using the full sample. The categories generated by the pilot analysis were regarded as preliminary and were later expanded and modified during the final analysis.

### Ethics

The study has been approved by the Swedish Ethical Review Authority (Dnr 2020-05096).

## Results

The analysis generated three themes and seven sub-themes, as presented in Table 2. We have chosen to assign theme names that reflect the children’s perspective. If there were writing in Swedish on the drawing, the English translation can be seen in the caption. Common motifs in the drawings were of how the children’s lives had changed, particularly by societal restrictions that impacted the children’s own lives. The children drew and wrote about big differences compared to their previous routines, and the changes described were almost exclusively negative, such as minimized mobility, cancelled activities, not seeing grandparents and other relatives, and restrictions within schools. Children further accurately depicted the virus particles, transmission routes, and how symptoms may vary within a population. The children also drew overcrowded hospitals and images representing death; how the disease spread across the world, covering news broadcasting, and even restrictions in countries outside of Sweden. This age group had a clear understanding of contrast, for example, the “before” versus the “now” of the pandemic, but also dystopic fantasies of the “after.”

### Theme 1: Fun, Friends, and Freedom Are Cancelled

In this theme, the children showed both their understanding of societal restrictions and recommendations in Sweden and in other countries, and they further strongly illustrated how these affect their own lives.

Under the sub-theme *(strong) reactions to prohibitions*, the children drew and expressed anger about not meeting others and resentment of having to stay at home more. Not seeing grandparents was one of the most frequent motifs and discussion points for the children where frustration and anger dominated the results. Grandparents were often illustrated with either a barrier between them and the child, or a large X drawn over a social gathering with grandparents or an X drawn over the grandparent alone. Adjoining texts further highlighted these kinds of illustration as can be seen in the quote below.

I want Corona to die. I didn’t get to see my grandma and grandpa. I don’t like Covid-19.

**Figure 2.**
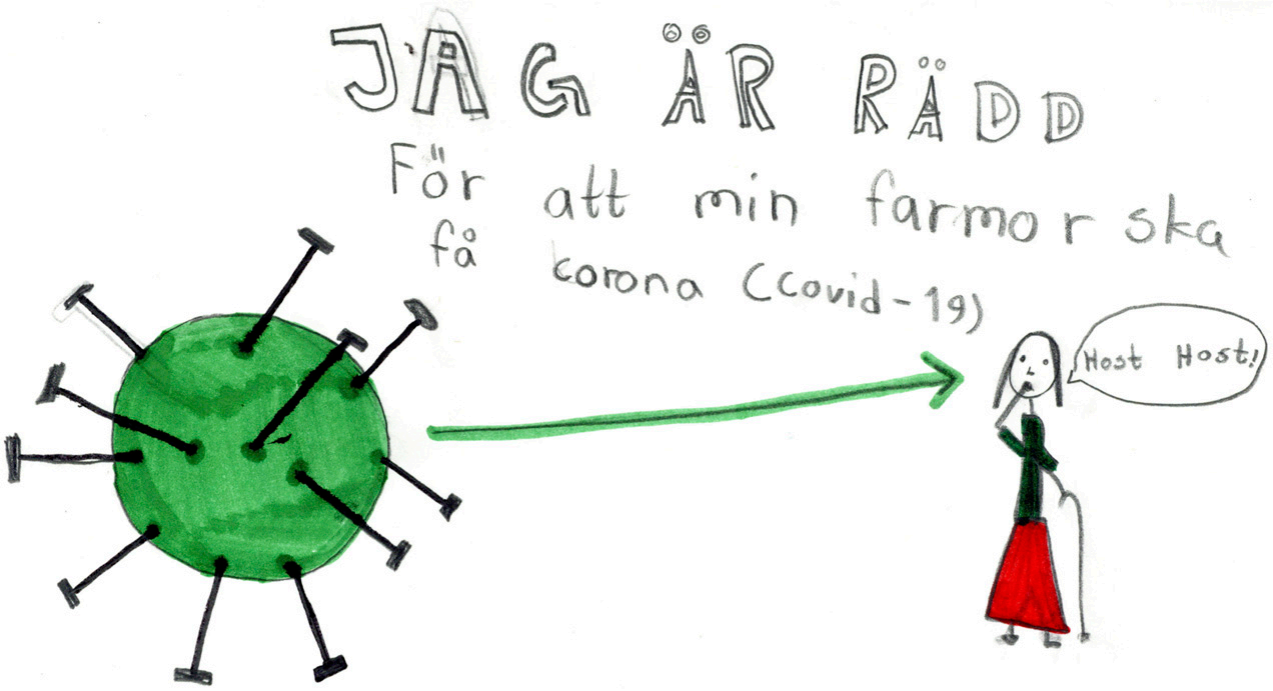
“I am afraid that my grandmother will get Corona (COVID-19) *caugh chaugh*.” Picture: 2020:009:0605. ©Swedish Archive of Children’s Art.

A few drawings also included feelings of sadness and worry (see Figure 2). Similar feelings were expressed toward grandparents dying or becoming ill from the virus, such as in Figure 2, with a massive virus particle in green with an arrow pointing toward a grandmother who is coughing.

**Figure 3.**
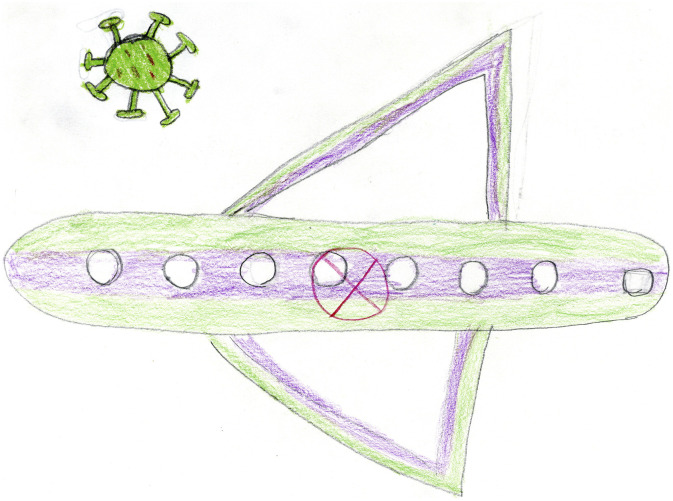
Picture: 2020:009:0448. ©Swedish Archive of Children’s Art.

**Figure 4.**
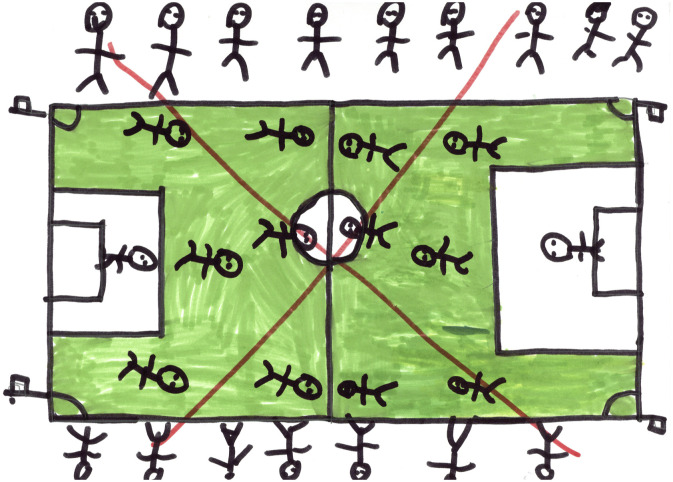
Picture: 2020:009:0276. ©Swedish Archive of Children’s Art.

In the sub-theme *(Strong) reactions to prohibitions*, cancelled holidays, trips, and activities were demonstrated by motifs with crossed-out aeroplanes (see Figure 3) or drawings depicting the travel destination, often illustrated as a beach with palm trees. But anger was also expressed toward no longer being able to take shorter domestic trips, shown particularly in the large red X drawn over many activities that were cancelled, as well as large exclamation marks on many drawings and in the texts. Cancellation of leisure activities included sports practices and matches, birthday parties, or dinners. Figure 4 shows a football pitch full of people and spectators that is crossed-out.

**Figure 5.**
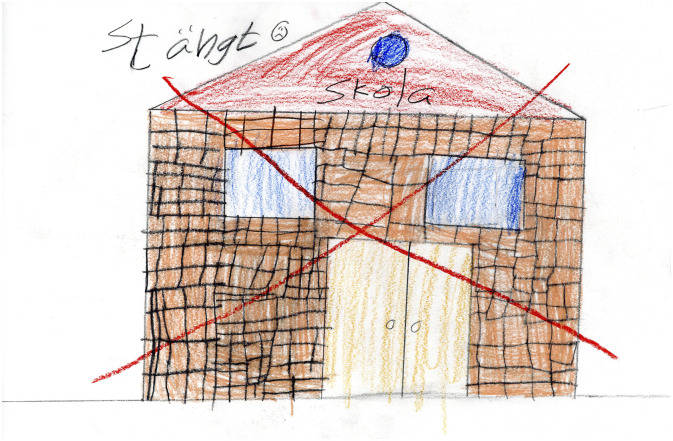
“Closed. School.” Picture: 2020:009:0579. ©Swedish Archive of Children’s Art.

Children’s drawings also related to school under this “cancellation” theme. Children expressed concerns about potential closure of their schools, as had happened for their older peers and for children in other countries (see Figure 5). Changes in schools also included things the children disliked:

“… at the school we are not even allowed to take the food ourselves [referring to self-service at lunch provided by all schools],” alluding to loss of serving oneself school lunch from the canteen, something children at this age start being able to do by themselves.

**Figure 6.**
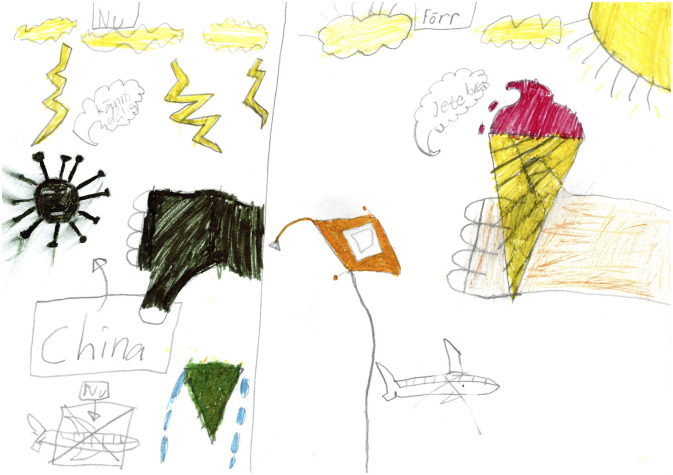
“Now, feels bad, China | Then, very good.” Picture: 2020:009:0116. ©Swedish Archive of Children’s Art.

Drawings of the sub-theme *contrasts* dealt with the contrasts between life before, during, and after the pandemic, almost exclusively drawn in an object perspective (a composition of different objects not necessarily a classic symmetry), a split image with a line down the middle illustrating different sides. The children reflected on what was good versus bad, at both micro and macro levels. Illustrations included contrasts in everyday life and how those differences affected the children themselves. Drawings of now/then and now/future (what’s to come) addressed contrasts between life now, during the COVID-19 pandemic, and then, before the COVID-19 pandemic, or the future after the pandemic. In Figure 6, the colors used in “now” (during the pandemic) are dark and desolate: black and green together with lightning from the sky and a crossed-out aeroplane. The right side of the drawing, representing the time before the pandemic, is in vibrant colors with a kite flying, an ice-cream, and an aeroplane, while the sun is shining down. These types of color differences were common throughout the sub-theme where dark colors often black, red, and green were shown in negative connotations.

The children also included drawings of the contrast that COVID-19 can be good for the environment but bad for people. Drawings focused heavily on depicting nature, with fish swimming in a clear blue ocean and bids flying above, using light and vibrant colors. This was specifically linked to the positive impact on the environment and the climate due to reduced emissions as a result of less travelling and mobility, which could be seen through empty streets and crossed-out cars and aeroplanes, and by texts reinforcing this.

Children also addressed contrasts between Sweden and other countries. Children described that many people abroad were in quarantine and not allowed to go to shops or meet friends at all. They further reasoned that it was not as bad in Sweden, and that here you only needed to wash your hands more and that certain activities were cancelled.

### Theme 2: The world Is All Upside Down

**Figure 7.**
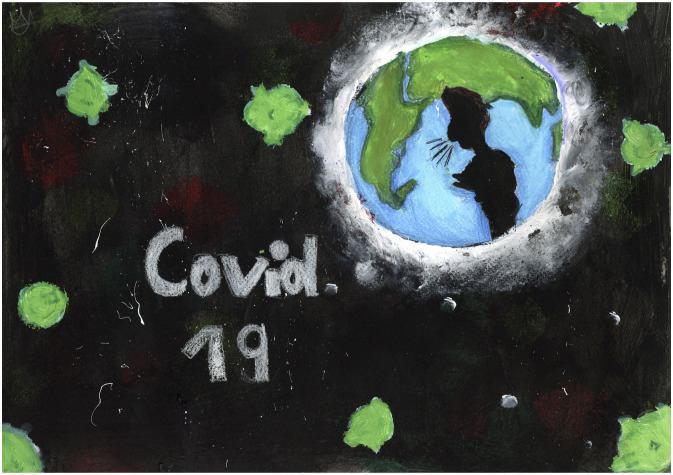
“COVID-19.” Picture: 2020:009:0490. ©Swedish Archive of Children’s Art.

Under the sub-theme *the whole world is affected*, children’s drawings cover that COVID-19 was in all the countries of the world, where it originated, and how it spread. Figure 7 shows how the COVID-19 virus had spread across the globe, and even into space. The children also depicted themselves as part of this world, and global individuals, sometimes bringing to light slight optimism, such as “we are all in this together” and illustrations of people holding hands.

The children also illustrated news reports about the spread and restrictions both in Sweden and abroad, the drawings mainly illustrated TV broadcastings and radios. News was depicted in a positive way, for example, in the children’s texts: “Covid-19 is bad. News is good” and “Good that they talk about it.”

**Figure 8.**
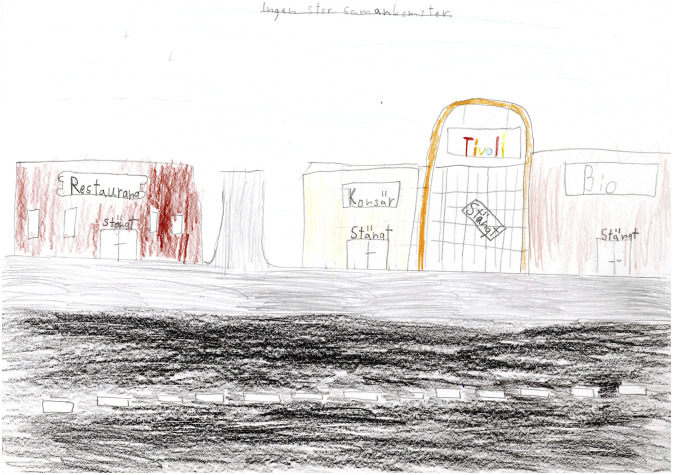
“No big gatherings. Restaurant, closed; Painter, closed; Tivoli, closed; Cinema, closed.” Picture: 2020:009:0149. ©Swedish Archive of Children’s Art.

The sub-theme *existential thoughts* was related to uncertainty and negative thoughts about the future, although a few positive and optimistic thoughts were present. Above all, there were dystopian thoughts about how the pandemic had affected society as well as fears for the future, and a fear if humanity would survive. This was depicted in illustrations that showed a society with empty streets, closed shops, and boarded up windows and doors, as in Figure 8 from a central perspective. In these drawings, colors were almost exclusively dark and the drawings were generally desolate with no depictions of living humans in them.

The children also showed fear for the end of the world, and of COVID-19 destroying the Earth, where the COVID-19 virus often appeared as a creature or monster, often large and either red or black that destroys or even eats large chunks of the Earth.

Drawings of full/overcrowded hospitals illustrated how the healthcare system became overloaded during the pandemic. Drawings often included long, winding queues from the hospitals and healthcare staff in protection suits (which can be seen in Figure 12), and the children expressed concerns that the difficulty to accommodate all patients may lead to death, both from COVID-19 and from other health issues:

Full in the hospitals. Many lose 70+ whom they love.

Further drawings included skulls and bones, crossed-out people, and black illustrations of humans lying with red blood streaming from them.

### Theme 3: The Virus: Evil, Scary, and Dangerous, but Masks and Sanitizers can Help

**Figure 9.**
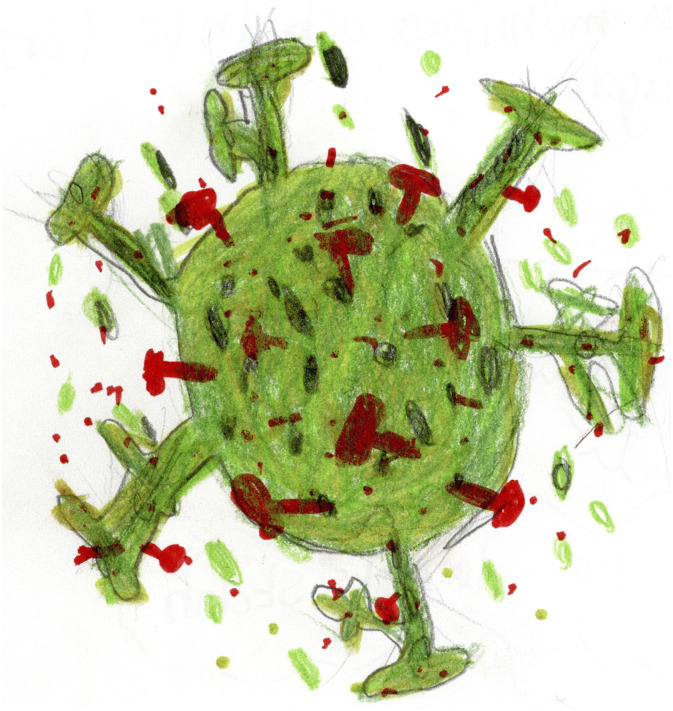
Picture: 2020:009:0436. ©Swedish Archive of Children’s Art.

The sub-theme *children’s depictions of COVID-19* was one of the most common motifs in the children’s drawings, where the virus was made real through illustrations of virus particles and creatures. The drawings included detailed illustrations of COVID-19 virus particles and different ways in which children thought the COVID-19 virus looked like. The virus particles (see Figure 9) were generally drawn from a value perspective, where the virus took center stage as large circular objects, centered on the page with the classic crown-like casing. Virus particles were most commonly drawn in green, but also red, black, and purple. In some instances, the particles could also appear on the sides of illustrations such as in Figures 2 and 14.

**Figure 10.**
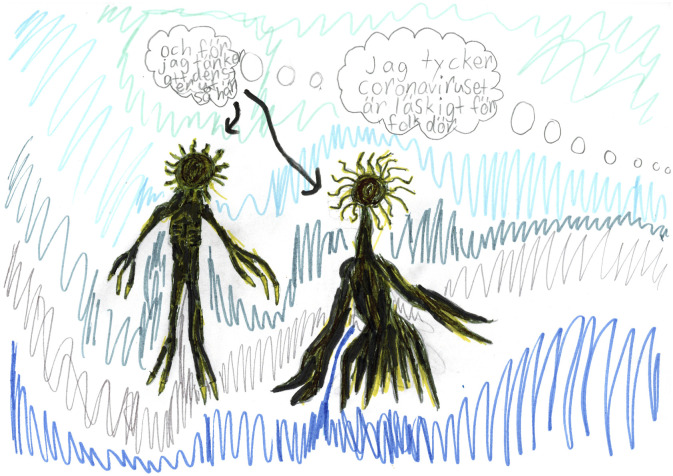
“I think the Coronavirus is scary because people are dying. And because I think it looks like this.” Picture: 2020:009:0139. ©Swedish Archive of Children’s Art.

Children also often drew the virus as a creature which included elements of anthropomorphism, fantasy, or aliens (Figure 10) where the virus is a creature with long tentacle-like bodies, arms, and legs, while the head still shows clear “realistic” influences of the virus’ crown-like envelope with projections, once again also illustrated in black and green.

**Figure 11.**
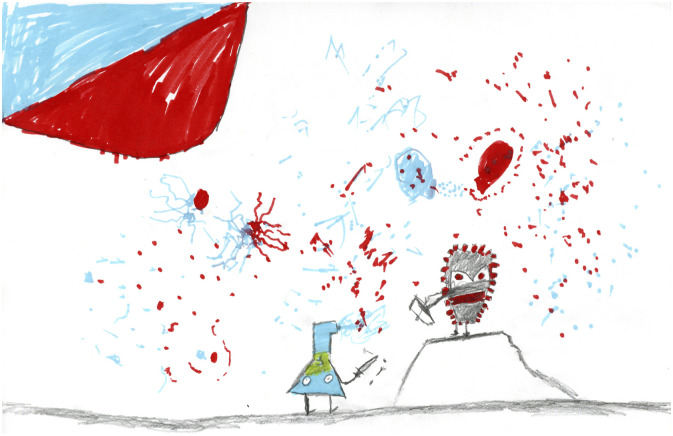
Picture: 2020:009:1091. ©Swedish Archive of Children’s Art.

With COVID-19 being illustrated as an evil creature, the children drew battles between the virus and humans, or other objects. The predominant color was red and black for the virus, illustrating these dark colors as “bad” or “evil,” while the hand sanitizer was depicted in a lighter blue. Throughout the image, specs of virus and hand sanitizer are fighting. In Figure 11, “a battle between the coronavirus and hand sanitizer” can be seen.

**Figure 12.**
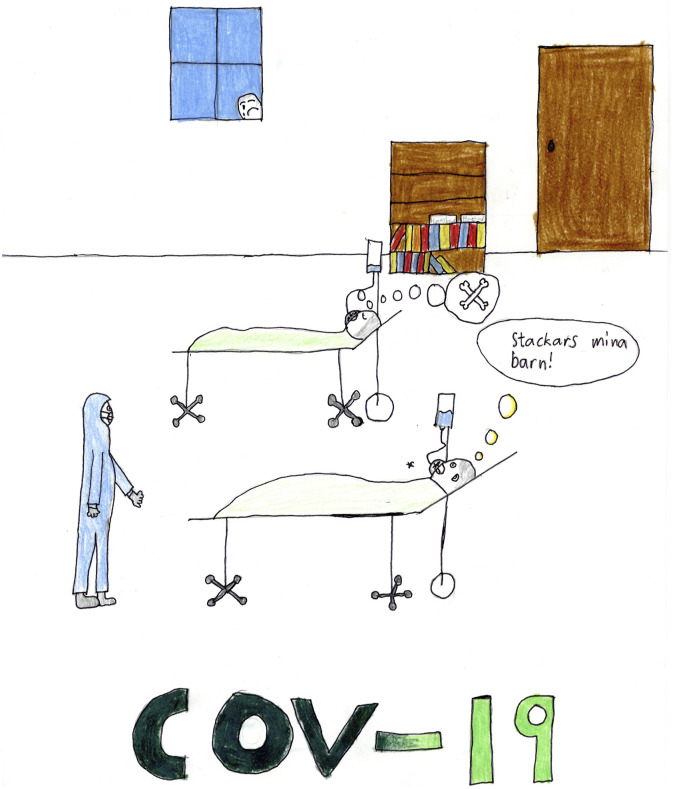
“My poor children. COVID-19.” Picture: 2020:009:0492. ©Swedish Archive of Children’s Art.

In the sub-theme *COVID-19 leads to illness and death*, the children drew how the virus spreads from person to person, its symptoms, and how the illness varied from mild to severe and in some cases even death. Death was further not an uncommon motif in the drawings. Two different aspects of death were illustrated: both dead people and that COVID-19 brings death with it, illustrated by Figure 12 with sick motionless people in hospital beds, personnel in protective gear, and a crying visitor who cannot enter the room.

Some children also drew that they had to attend funerals digitally, sadness in the form of crying people, and fear that their parents or other relatives would get sick and die.

Children also illustrated symptoms by drawing people with fevers and impaired lung-function. Drawings of the illness often included green snot dripping from a person’s nose and green drops coming out of mouths while coughing; illustrations of lungs varied from healthy pink to darker black. Transmission was commonly illustrated by two people standing close together while drops of liquid from one hit the other. Children also drew vaccines, often liquid filled syringes, and wrote that they wished there was a vaccine, or a cure as they phrased it themselves. In the drawings collected toward the end of the data collection period, the children described positive thoughts on the development of vaccines.

**Figure 13.**
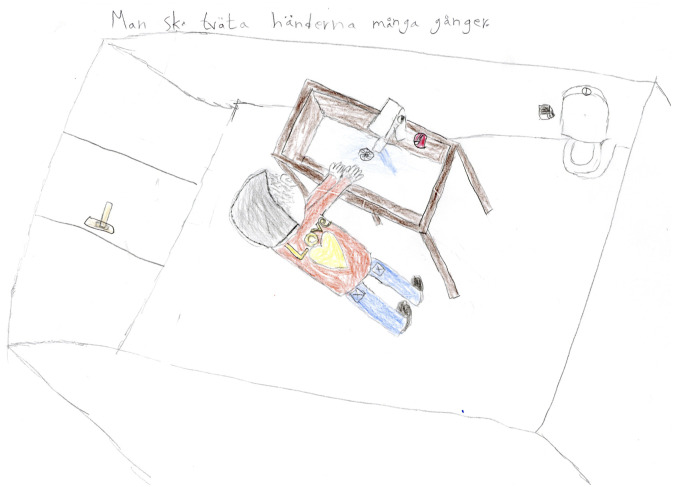
“You should wash your hands many times.” Picture: 2020:009:0151. ©Swedish Archive of Children’s Art.

Under the sub-theme *means of protection and reduction of transmissions*, various measures to control and reduce the spread of infection while protecting the individual were depicted. There was a strong focus on hand hygiene, which included illustrations of hand-washing with soap and illustrations of hand sanitizers, with texts encouraging people to maintain the recommended hand hygiene procedures (see Figure 13). Drawings of face masks showed how to protect oneself from being infected by the COVID-19 virus with the use of masks, despite face masks not being mandatory in Sweden.

**Figure 14.**
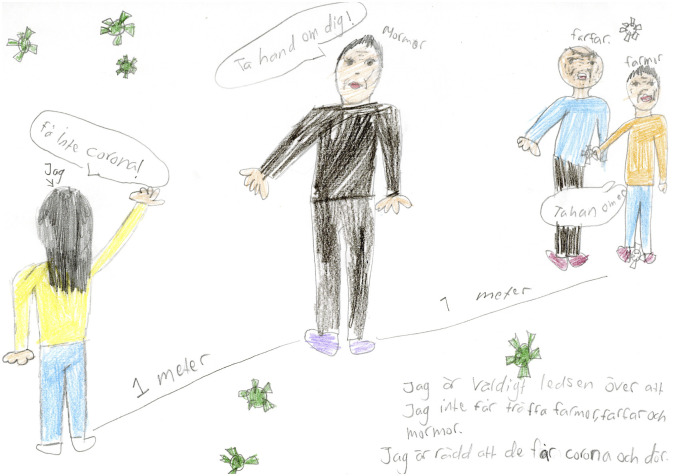
“Me; do not get Corona! 1 meter. Grandmother; take care of yourself! 1 meter. Grandmother & grandfather; Take care of yourself. I am very sad over the fact that I cannot see my grandparents. I am afraid they will get Corona and die.” Picture: 2020:009:0548. ©Swedish Archive of Children’s Art.

Children drew the importance of maintaining social distancing (Figure 14), and that this along with staying home if you are having symptoms or feeling ill reduces the spread of COVID-19. The children drew lines between people with indication of how far apart you needed to remain (Figure 14). They also expressed sadness about not being able to hug, by drawing crossed-out hugs.

The drawings also illustrated alternative ways of socializing during the pandemic such as being outdoors and connecting via technology. Socializing outdoors, often depicted as playing outside, was generally depicted as something positive by the children, since this meant they could still meet, but it was also viewed with some reluctance. Socializing via technology was another way for children to stay in touch with grandparents. Drawings mainly contained motifs of phone calls and video calls. There was a certain frustration due to the lack of meeting physically, but video calls were still seen as a viable option. Finally, children drew pictures of parents working from home offices. The reasoning of the children varied between parents working at home to avoid being infected by COVID-19, to avoid spreading COVID-19, and/or due to restrictions and recommendations from authorities, conveyed through news.

## Discussion

This study is based on semiotic visual and content analysis of 454 drawings and included texts by Swedish 7- to 11-year-olds, collected during the first wave of the COVID-19 pandemic. The study has provided a unique insight into Swedish children’s knowledge and experiences of the COVID-19 pandemic. Through the analysis process, we identified three main themes: *Fun, friends, and freedoms are cancelled*, which pointed to societal changes as a result of the pandemic that impacted the children’s own lives, causing high levels of frustration; how they reflected on this and saw contrasts between what had been, what is, and what is to come. *The world is all upside down*, in which the children highlighted their understanding of the virus, how it has changed and impacted the world, as well as existential reflections of their lives and surrounding environment. *Virus: evil, scary, and dangerous, but masks and sanitizer* *can help* showed the children’s high level of health literacy related to COVID-19.

### The Knowledgeable and Well-Informed Child and Global Citizen

In the international literature, negative feelings related to societal restrictions and their implications were more related to fear and sadness (Bonoti et al., 2021; Cornaggia et al., 2022; Idoiaga et al., 2020; Idoiaga Mondragon, Eiguren Munitis, Berasategi Sancho, & Ozamiz Etxebarria, 2022; Sarkadi et al., 2022). Interestingly, the children in this study often expressed frustration and anger as a response to these restrictions, which was a unique finding compared to existing literature, even compared to age groups. Frustration and anger were often shown through dark colors, composition and perspective of the drawing, hard pressure of pen strokes, and by the texts. Much frustration was centered around cancelled holidays and activities as well as reduction of autonomous activities, such as not being able to serve themselves from the free lunches provided to children in all Swedish schools. These frustrations were reflected in the virus taking center stage in the composition being depicted with strong colours and as disproportionately large and described as “hard,” “stupid,” “mean,” and “not good.” Anger and frustration were more prominent than sadness and fear, although these emotions were also present, often drawn through crying or sad faces.

Despite that it is quite uncommon in Sweden to cohabit with extended family (StatisticsSweden, 2024), such as grandparents, the study’s finding of children’s extremely strong negative emotions (crying, exclamation marks, crossed-out social gatherings) toward not seeing grandparents shows that this connection is hugely important and meaningful for the children, possibly more than the very modern, secular, individualistic Swedish society had assumed or understood beforehand.

The children’s concern for the school closing (shown through crossed-out school buildings with a sign “closed”) reflected their awareness of what was happening to secondary school students and how this impacted them, as well as that many children were affected by school closures in other parts of the world. The Swedish response to COVID-19 was keeping schools open for younger children, which in this sample showed to be extremely valuable. While the open policy Sweden maintained during the pandemic was criticized across the world, for children in this sample, it seemed very important to keep schools open and remaining some form of normalcy. In hindsight, it was also a very wise decision, both in terms of prevented educational loss and infection control (Björkman et al., 2023).

The children in this study presented themselves as global beings, stating “we are all in this together.” The children had a good understanding of news feeds, what recommendations apply to different groups, and when they were introduced, similar to findings from a UK study using interviews and drawings, looking at the same age group (Thompson et al., 2021). In depictions of news and information of the virus spreading across the world, the children followed the development in other countries. For example, there were drawings alluding to high number of fatalities in countries like the United States and that people had to be in quarantine in certain parts of the world. Summarizing their impressions from media and societal discourse, children depicted the Earth being destroyed by the pandemic. This attention to the news can also be a result of the time and a reflection of the social dynamic from surrounding adults, both in school and from parents or caregivers.

The children also demonstrated a high level of health literacy related to COVID-19, similar to a study on 4- to 6-year-olds and on 13- to 15-year-olds (Sarkadi et al., 2021; Thompson et al., 2021). Similarities in the illustrations of the appearance of the virus can be seen in these results and in the Spanish studies (Bonoti et al., 2021; Bray et al., 2021; Idoiaga Mondragon, Eiguren Munitis, Berasategi Sancho, & Ozamiz Etxebarria, 2022), showing that the communication around the virus, its spread, and containment measures was powerful and reached children from a young age. Reducing transmission through social distancing, hand hygiene, and staying at home if you are sick is in line with studies on children’s perceptions of health and illness (Pridmore & Bendelow, 1995).

### Implications for Crisis Management and Communication

This research continues to add insights to the growing body of literature on children in disasters and crises, where the results show that even young children have a high understanding of health, disease, and societal responses to these. The results were further in line with Ribeiro and Silva’s study where children showed that they were capable and to a large extent preferred being active agents in crises and disasters (Ribeiro & Silva, 2020). The Swedish children’s drawings showed that despite their young age, children could reason about characteristics of the disease, how it looked and spread, as well as understanding and following societal recommendations and regulations. They could reason outside of themselves and reflect on the impacts on society, and in fact, the world, as a whole. Of special interest is 7- to 11-year-olds’—sometimes dystopic—thoughts and concerns regarding the future. It is important to address these concerns as part of the crisis response, as children lack the ability to put things into perspective. The fact that so many of these children felt frustrated by the imposed restrictions and loss of autonomy may also have to be addressed in crisis communication by normalizing these feelings and finding constructive outlets so children can contribute and feel less disempowered. Encouraging children to draw their thoughts and feelings and then discuss these with them is a useful gateway into children’s worlds and a possibility to address their concerns.

### Methodological Discussion

The fact that the method allowed for children to freely illustrate their thoughts about the virus, its appearance, how it has affected society, and how it feels provided a unique insight into the children’s understanding and world. This is a strength of the method of analyzing children’s drawings (Aronsson, 2022). The children, in an autonomous way, conveyed what they themselves considered most important about the pandemic and the COVID-19 virus. The drawings also allowed for an analysis of what and how much of the surrounding world the children pick up, understand, and interpret.

A potential weakness of the method is the possibility of misunderstanding or misinterpreting the drawings. We have tried to minimize this risk by using analysis methods based on established and carefully tested research on children’s images—content and visual and semiotic analysis (Aronsson, 2022; Graneheim & Lundman, 2004; van Leeuwen & Jewitt, 2001). The accompanying texts were also used as “pointers,” to provide deeper understanding of the drawings, and drawings without text were not included in the analysis to avoid misinterpretation.

Another potential weakness of the study is the lack of representation of different groups, particularly the lack of background variables. We chose to include all drawings with relevant age and accompanying text. Children who were unable to or unwilling to express themselves in text (self-written or dictated to an adult) were therefore not included. We also know that adult participation was needed to inform the children about the data collection for SBBA and that it was probably committed educators, leisure leaders, or parents who encouraged the children to draw and then sent in the drawings. We also do not have any information about the instructions given, although the questions to be answered were clearly stated in the SBBA invitation. Future research could benefit from including children in the interpretation and analysis process of the drawings.

Furthermore, the children received four questions in the call from SBBA, as stated in the Methods section. These questions were meant to help inspire or get the children started with drawing their thoughts and experiences of the virus; however, this has most likely also shaped the results in accordance with the posed questions.

Negative drawings of the virus and its characteristics and negative reflections on prohibitions as consequences of the pandemic dominated the results. This is more likely a reflection of the fact that they are consumers of the world around them, through media, schools, and parents, rather than the questions posed per se.

We have some information about the children’s geographical affiliation, and there is a reasonably large spread across Sweden, ranging from high income municipalities with a relatively homogeneous population to municipalities with below national average median income, and municipalities with a more diverse population. However, since the data was used from an archive, we have no specific information about the individual children’s demographic profiles. Thus, the sample in this study cannot be considered representative of children aged 7–11 in Sweden, nor can it reveal whether the pandemic has had an uneven impact on children from a socio-economic perspective. Hence, future research would benefit from focusing specifically on children with a more vulnerable demographic background which would be beneficial to compare to the results of this specific study to see how it may differ.

Further, the method of analyzing children’s drawings is found to be both feasible and appropriate given the sample size, and we argue that it should be included more in research focusing on children. Including children’s own voices and expressions in research gives further nuance to results and highlights perspectives that adults may not be considering. This is a strong argument for continuing using child-centered methods in research, and for them to be seen as autonomous actors with the ability to make conscious decisions about themselves and the world around them.

## Conclusions

The study has shown that children had in-depth knowledge and understanding of the COVID-19 pandemic, from the appearance of the virus and how the disease affects people, to the societal effects of the pandemic. Children’s main concern was the potential illness and death of grandparents whom they missed seeing. A novel finding was that the emotion that dominated the drawings of this age group was not fear or anxiety, as seen in other studies from countries with major lockdowns and school closures, but frustration. The findings also highlight children’s broad understanding of the pandemic as a global event with implications for people and societies globally, both now and in the future. Despite Sweden’s relatively lenient measures, the pandemic significantly affected their lives and autonomy.

### Significance

We are living in a time when societal crises seem to follow each other. Children aged 7–11 years show a very good understanding of what is going on in their environment, take part in the news, and are affected by adults’ concerns. It is therefore important that research, responsible authorities, non-governmental organizations, and the media account for children’s need for information and how children are affected in their daily lives by a current societal crisis and disasters. Children use their imaginations to understand things that may be difficult to comprehend, become frustrated at being restricted in various aspects of their daily lives, but can also be involved in implementing the measures that society has decided are necessary, as was seen in Ribeiro and Silva’s (2020) study, where children felt better and useful when included in the work against the disaster. Using accessible language and also encouraging children’s own creativity can help to empower children and address their concerns and frustrations during crises. This should be seen as an important part of society’s sustainable crisis preparedness.
